# Report of July Meeting

**Published:** 1861-08

**Authors:** John Wright

**Affiliations:** Secretary, pro tem.


### DEWITT COUNTY MEDICAL SOCIETY.

This Society met in Odd Fellows Hall, Marion, July 2, 1861; the President, Dr. Madden, in the chair. The meeting was opened with prayer by Rev. M. Richards, after which the minutes of the previous meeting were read and approved.

Drs. D. W. Edmiston, R. T. Richards and B. K. Shurtleff made application for membership. The censors examined the applicants, reported favorable to, and recommended, their election. On motion of Dr. Lewis the report was received and the candidates unanimously elected members of the society. Society adjourned to meet at 2 o'clock, p. m.

Afternoon Session.—Dr. Tyler reported an interesting case of what was called pneumonia, by a so-called eclectic doctor of Wapella. The disease, however, proved to be an affection of the throat. The case proved fatal, probably from the error in diagnosis. Dr. Tyler also presented an interesting case of cardiac affection, which was examined by the members of the Society. The case elicited a lengthy discussion, in which all the members present participated.

Diptheria, the subject for general discussion was taken up, but for the want of time was cut short. Dr. Wright gave notice that he would read a paper on diptheria, at the next meeting of the society.

Drs. Edmiston and Adams, the regular essayists, were not present. Dr. T. W. Davis was also expected to read a paper, but did not. He was excused. Drs. Edmiston and Adams were continued. Dr. Richards was also appointed to read an essay at the next meeting.

On motion, the thanks of the society were tendered to the Odd Fellows, for the use of their hall. On motion, the thanks of the society were tendered to Dr. Tyler and lady, for the excellent dinner gotten up, at their expense for the society. On motion, the proceedings of this meeting were ordered to be published in the *Central Transcript* and the Chicago medical journals.

On motion, the society adjourned to meet in Mount Pleasant the first Tuesday in October next, at 10 o'clock, a. m.

JOHN WRIGHT, *Secretary, pro tem.*

